# Dynamic alteration in the gut microbiota and metabolome of Huanjiang mini-pigs during pregnancy

**DOI:** 10.1186/s12917-022-03477-0

**Published:** 2022-11-03

**Authors:** Peifeng Xie, Chengjun Hu, Md. Abul Kalam Azad, Qinghua He, Qian Zhu, Xiangfeng Kong

**Affiliations:** 1 Key Laboratory of Agro-Ecological Processes in Subtropical Region, Hunan Provincial Key Laboratory of Animal Nutritional Physiology and Metabolic Process, National Engineering Laboratory for Pollution Control and Waste Utilization in Livestock and Poultry Production, Institute of Subtropical Agriculture, Chinese Academy of Sciences, 410125 Changsha, China; 2 Tropical Crop Genetic Resource Research Institute, Chinese Academy of Tropical Agricultural Sciences, 571101 Haikou, China; 3 Department of Food Science and Engineering, College of Chemistry and Environmental Engineering, Shenzhen University, 518060 Shenzhen, Guangdong China

**Keywords:** Gut microbiota, Huanjiang mini-pigs, Metabolites, Fat accumulation, Pregnancy

## Abstract

**Background:**

Maternal gut microbiota and metabolites are associated with their offspring’s health. Our previous study showed that maternal body fat percentage increased from days 45 to 110 of gestation in a Huanjiang mini-pig model. Thus, this study aimed to investigate the changes in gut microbiota composition and microbial metabolite profile of sows from days 45 to 110 of gestation.

**Results:**

Twenty-four Huanjiang mini-pigs with average body weight were assigned for sample collection during early- (day 45 of pregnancy), mid- (day 75 of pregnancy), and late-pregnancy (day 110 of pregnancy). The results showed that the relative abundances of *Clostridium_sensu_stricto*_*1*, *Romboutsia*, *Turicibacter*, and *Streptococcus* in jejunal contents were higher at day 110 than those at day 45 or 75 of gestation. In the ileum, the relative abundance of *Streptococcus* was higher (*P* < 0.05) at day 110 of gestation, as well as the metabolism function of the jejunal and ileal microbiota. The ileal butyrate and acetate concentrations were higher at days 45 and 110 of gestation, respectively. In the colon, the concentrations of cadaverine and spermine were higher (*P* < 0.05) at days 45 and 110 of gestation, respectively. Metabolomic analyses demonstrated that the metabolic pathways, including D-glutamine and D-glutamate metabolism, phenylalanine/tyrosine/tryptophan biosynthesis, and alanine/aspartate/glutamate metabolism changed during gestation.

**Conclusion:**

Collectively, our results showed that gut microbiota composition and microbial metabolites changed dramatically from early to late pregnancy in a Huanjiang mini-pig model. These findings will provide new targets in formulating maternal nutritional interventions to alleviate the adverse effects during pregnancy on offspring health outcomes.

**Supplementary Information:**

The online version contains supplementary material available at 10.1186/s12917-022-03477-0.

## Background

Maternal physiological and biological changes during pregnancy can highly influence the growth and development of conceptus, resulting in long-term health problems in the offspring [1]. Gut microbiota impacts body physiology and is associated with the etiology of various diseases, including obesity, type 2 diabetes [2], and insulin resistance [3]. Evidence showed that maternal metabolism was associated with the changes of gut microbiota composition [4]. In addition, pregnancy metabolic syndrome is associated with the changes of gut microbiota composition in the third trimester of pregnancy [5]. These findings indicated that gut microbiota presented an important role in maternal metabolism during gestation.

Intestinal microbiota shapes host physiology through their metabolites. These metabolites, such as short-chain fatty acids (SCFAs) and bioamines, play important roles in host physiology [6]. The SCFAs are mainly produced by colonic microbiota from dietary carbohydrates and proteins [7] and are associated with metabolic syndromes [8]. For instance, butyrate protects against diet-induced fat accumulation by increasing energy expenditure [9], and acetate mediates a microbiome–brain–β-cell axis to promote metabolic syndromes [10]. Therefore, evaluating the influence of the microbiota composition on host metabolism will help to reveal the relationship between gut microbiota, metabolites, and their host.

Intestinal microbiota and metabolites play important roles in different physiological states during pregnancy. Research evidence showed that the composition of gut microbiota and metabolites are inconsistent in mammals during pregnancy [11]. However, changes in intestinal microbiota and metabolites during different stages of pregnancy are still unknown. Pig has served as one of the animal models in clinical medicine applications on human pregnancy [12]. Therefore, we used 16 S rRNA sequencing and metabolomics technologies to investigate the gut microbiota composition and identify microbial metabolite markers from days 45 to 110 of gestation in a pig model.

## Materials and methods

### Animals and housing management

A total of 24 primiparous Huanjiang mini-pigs with average initial body weight (BW) of 30 kg were obtained from a pig farm located in Huanjiang County, Guangxi Province, China. The sows were randomly assigned to one of eight pens, with three sows per pen. The animals were fed a diet formulated according to the recommendations of the Chinese National Feeding Standard for Swine (Additional file 1: Table S1). All animals were housed in 2 × 3 m pens with cement-sclerified flooring. Each pen was equipped with a feeder and a nipple drinker. All sows had *ad libitum* access to drinking water and were fed three times daily (about 2% of BW) [13].

### Sample collection and preparation

At days 45 (early-pregnancy, *n* = 8), 75 (mid-pregnancy, *n* = 8), and 110 (late-pregnancy, *n* = 8) of gestation, sows were euthanized using electrical stunning (120 V, 200 HZ) and exsanguination. The jejunal contents (middle portion), ileal and colonic luminal contents from a region 10 cm anterior and posterior to the ileo-cecal valve were collected, respectively [12, 14]. All samples were collected into sterile tubes and stored at −80 °C for further analysis.

### DNA extraction and PCR amplification

The total genomic DNA was extracted from the intestinal content samples using HiPure Stool DNA Kits (Magen, Guangzhou, China) according to the manufacturer’s instructions. The concentrations of extracted DNA were measured using a NanoDrop ND-1000 spectrophotometer (NanoDrop Technologies Inc., Wilmington, DE, USA). The total DNA of the intestinal contents was diluted to 50 ng/µL and then used to prepare amplicons for high-throughput sequencing.

The V3−V4 hypervariable regions of the bacterial 16 S rRNA gene were amplified as described previously [14]. The PCR reactions volume (10 µL) comprised 4 µL of 5×FastPfu Buffer, 2 µL of 2.5 mM dNTPs, 0.8 µL of each primer (5 µM), 0.4 µL of FastPfu Polymerase, and 2 µL DNA. The PCR reactions were conducted using the following program: 3 min of denaturation at 95 °C; 27 cycles of 30 s at 95 °C, 30 s for annealing at 55 °C, and 45 s for elongation at 72 °C; and a final extension at 72 °C for 10 min. The PCR products were extracted using 2% agarose gel, further purified using the AxyPrep DNA Gel Extraction Kit (Axygen Biosciences, Union City, CA, USA), and quantified using QuantiFluor™-ST (Promega, USA) according to the manufacturer’s protocol. Purified amplicons were operated using paired-end sequencing on Illumina MiSeq (Illumina, San Diego, USA). The instructions for the platform and manufacturer were from a commercial service provider (Majorbio, Shanghai, China).

### Processing of sequencing data

Raw fastq files were demultiplexed, quality-filtered by Trimmomatic (v.0.30), and merged by FLASH (v.1.2.11) with the following criteria: (i) the reads were truncated at any site receiving an average quality score < 20 over a 50 bp sliding window; (ii) primers were exactly matched allowing two nucleotides mismatching and reads containing ambiguous bases were removed; (iii) sequences with overlap > 10 bp were merged according to their overlap sequence. Operational taxonomic units (OTUs) were clustered with a 97% similarity cutoff using UPARSE (v7.1 http://drive5.com/uparse/), and chimeric sequences were identified and removed using UCHIME. The taxonomy of each 16 S rRNA gene sequence was analyzed by the Ribosomal Database Project Classifier algorithm (http://rdp.cme.msu.edu/) against the Silva (SSU123) 16 S rRNA database using a confidence threshold of 70% [15]. The ACE, Chao1, and Simpson indexes for microbiota were used to estimate alpha diversity values. The unadjusted means of OTU level microbial abundances were analyzed using partial least squares discriminant analysis (PLS-DA). The intestinal microbial functions were predicted by the phylogenetic investigation of communities by reconstruction of unobserved states 1 (PICRUSt1) analysis.

### Intestinal metabolites analysis

The SCFAs, including acetate, propionate, butyrate, iso-butyrate, valerate, and iso-valerate, were analyzed using gas chromatography (Agilent Technologies 1206, Santa Clara, CA, USA). Bioamines, including putrescine, cadaverine, spermidine, spermine, and tyramine, were measured using high-performance liquid chromatography (Agilent Technologies, Santa Clara, CA, USA) as described previously [13].

### ^**1**^ H NMR spectroscopy analysis

^1^ H NMR spectra were conducted according to the previous study [16]. Briefly, the 90° pulse length (~ 10.0 ms) was adjusted individually for each sample. The transients were collected into 32 k data points for each sample, with a spectral width of 20 ppm and a recycle delay of 2.0 s. The NMR spectral data scaled to unit variance were analyzed by the orthogonal projection to latent structure discriminant analysis (OPLS-DA) method. Principal component analysis (PCA) was performed to identify differential metabolites among the groups. Each metabolite was assigned for the variable importance in the projection (VIP) value according to the PLS-DA results. Metabolites with fold change VIP value > 1 and *P* value < 0.05 were used to select the significant differential metabolites between the three gestation stages.

### Statistical analysis

The alpha diversity indices of the intestinal microbiota communities and colonic microbial metabolites were analyzed using a one-way analysis of variance and Duncan’s multiple-range *post-hoc* test in SAS (SAS Institute, Inc., Cary, NC). The Student’s t-test was performed to compare the two groups. The relative abundances of microbial communities at the phylum and genus levels were analyzed using the Kruskal Wallis test. The correlations between microbiota and metabolic parameters were analyzed using Pearson’s linear correlation coefficient. Differences between means were considered to be statistically significantly different when *P* < 0.05, and 0.05 ≤ *P* < 0.10 were considered a trend.

## Results

### Body weight and diversity of the intestinal microbiota communities

The body weight of sows increased as gestation extended (Table 1). After size filtering, quality control, and chimera removal, 1,997,373 high-quality reads were obtained. The average read length was 440 bp. All samples were normalized, and the OTU table within each sample was rarefied to 22,701 sequence reads based on the sample rarefaction curves and Shannon curves (Additional file 2: Fig. S1). A Venn diagram was used to investigate the core microbiota presented in intestinal contents during different gestation stages (Additional file 2: Fig. S2). The results showed that 555, 321, and 984 OTUs were shared in jejunal, ileal, and colonic contents, respectively.

**Fig. 1:**
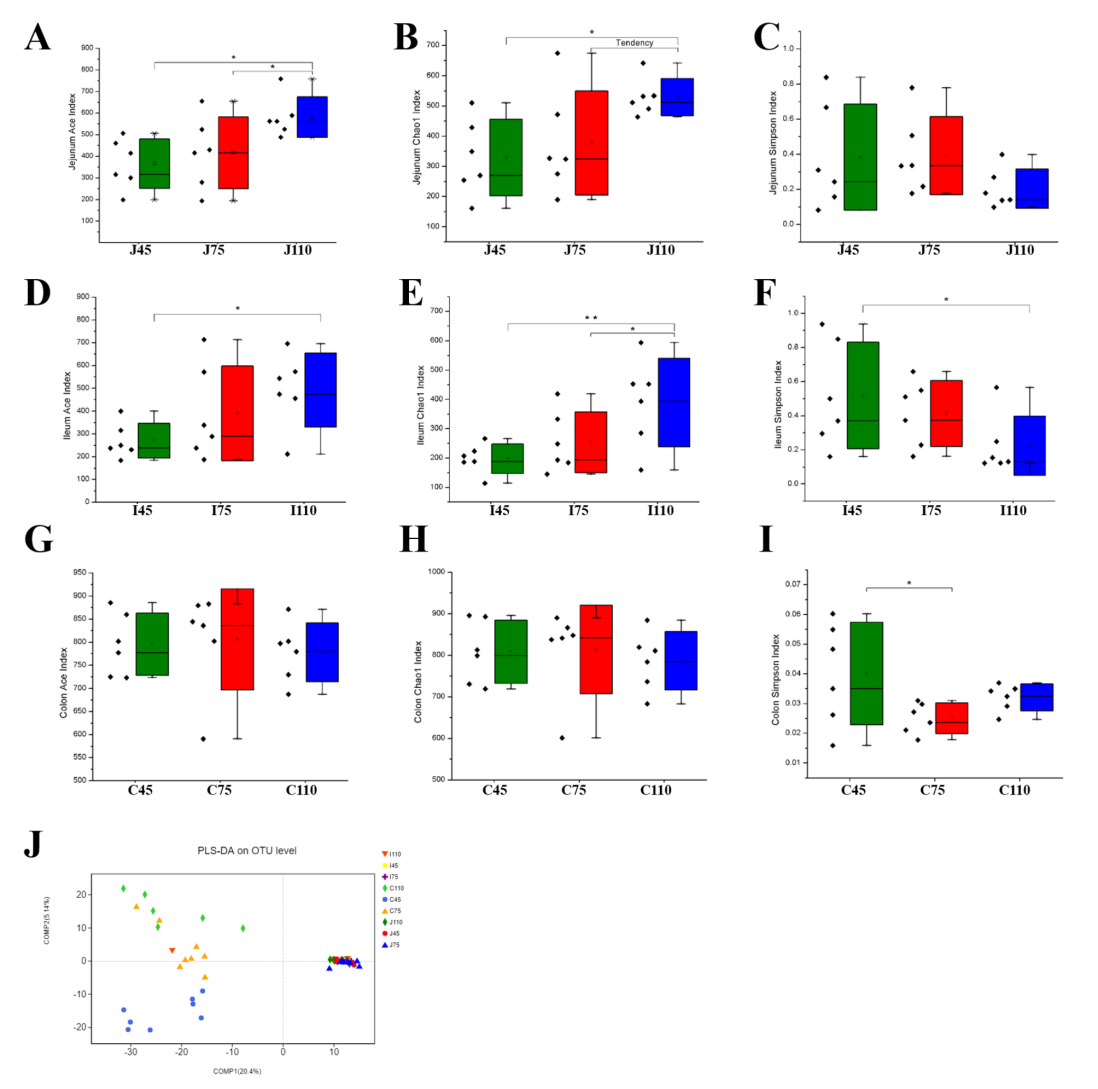
Alpha diversity indices of microbiota communities in jejunal, ileal, and colonic contents. (**A−I**) The microbiota diversity was estimated by ACE, Chao1, and Simpson indices, respectively. (**J**) PLS-DA of microbiota community. ^*^P < 0.05, ^**^P < 0.01; trend 0.05 ≤ *P* < 0.1. *n* = 7−8 per group.

The normalized sequence reads were used to calculate richness and diversity indices. As gestation extended, the ACE and Chao1 indices were increased (*P* < 0.05) in jejunal and ileal contents (Fig. 1A−F). In colonic contents, the Simpson index trended to decrease (*P* = 0.06) along with gestation extended (Fig. 1G−I).

The PLS-DA was used to analyze the differences among the three groups. As shown in Fig. 1J, samples from jejunal and ileal contents were clustered together. For colonic contents, the samples collected at days 45, 75, and 110 of gestation were separated from each other.

**Table 1:** Maternal body weight and the concentrations of short-chain fatty acids in the intestinal contents in different gestation stages

Items		Day of gestation		
		45	75	110
Maternal body weight (kg)		51.1 ± 3.42^c^	58.0 ± 2.02^b^	72.4 ± 3.66^a^
Jejunum	Acetate (mg/g)	0.60 ± 0.13^a^	0.43 ± 0.08^b^	0.53 ± 0.12^ab^
	Butyrate (mg/g)	0.14 ± 0.01	0.17 ± 0.04	0.14 ± 0.04
Ileum	Acetate (mg/g)	0.77 ± 0.15^b^	0.79 ± 0.10^b^	1.35 ± 0.42^a^
	Butyrate (mg/g)	0.21 ± 0.06^a^	0.10 ± 0.01^b^	0.14 ± 0.02^b^
Colon	Acetate (mg/g)	3.25 ± 0.85^a^	3.01 ± 0.85^a^	1.58 ± 0.41^b^
	Butyrate (mg/g)	0.70 ± 0.36	0.60 ± 0.20	0.55 ± 0.24
	Propionate (mg/g)	1.36 ± 0.33	1.35 ± 0.53	0.96 ± 0.59
	Valerate (mg/g)	0.11 ± 0.03	0.13 ± 0.03	0.10 ± 0.03
	Iso-butyrate (mg/g)	0.10 ± 0.03	0.10 ± 0.02	0.09 ± 0.01
	Iso-valerate (mg/g)	0.10 ± 0.06	0.11 ± 0.04	0.09 ± 0.02

Data are represented as means ± SD (*n* = 8). Different superscript lowercase letters within the same row indicate significant difference (*P* < 0.05).

### Composition of intestinal microbiota communities

**Fig. 2:**
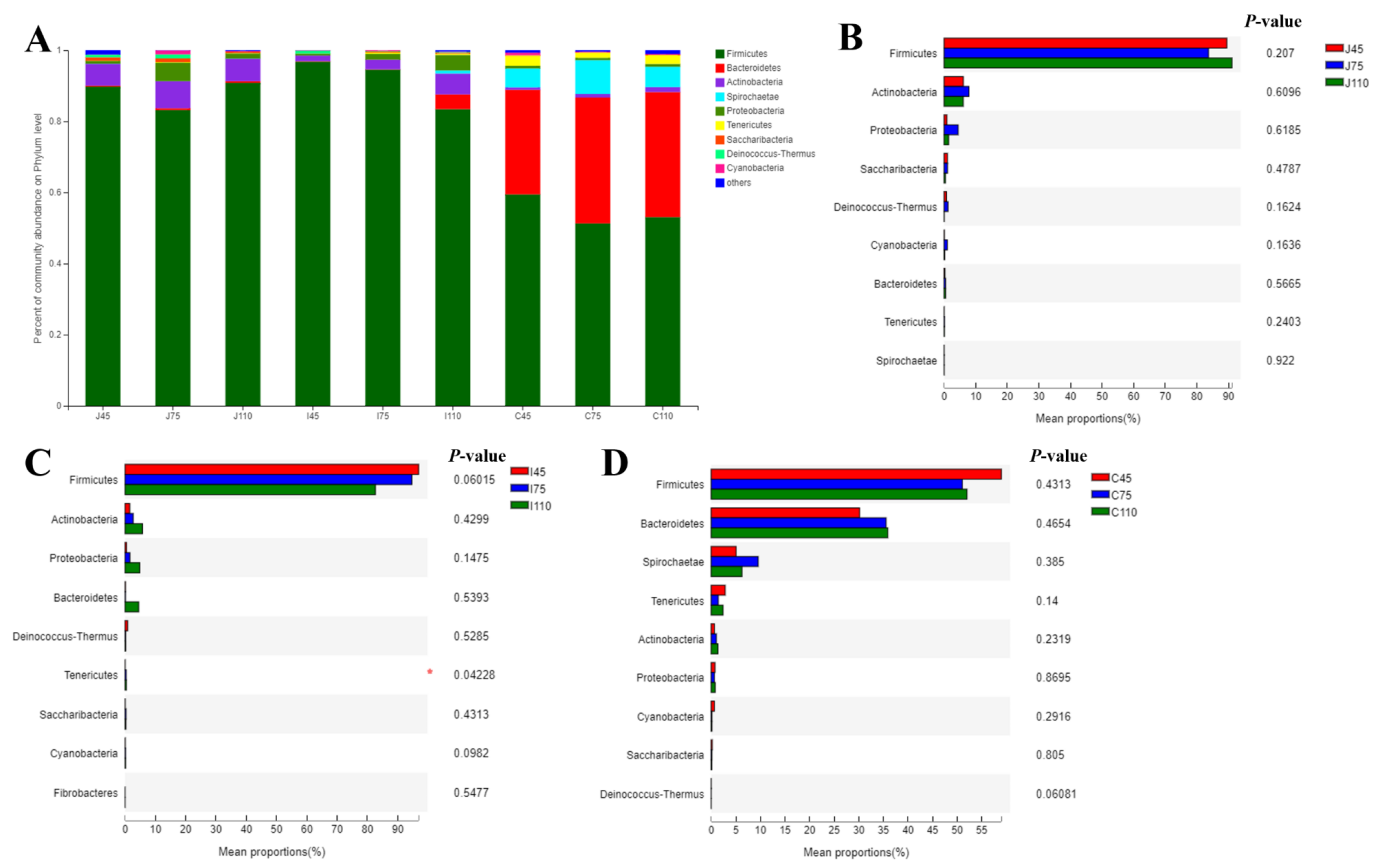
Microbiota communities at the phylum level. (**A**) Distribution of microbiota at the phylum level. Phyla with proportion < 0.01 were grouped in others. Comparison of relative microbiota abundance in jejunal (**B**), ileal (**C**), and colonic (**D**) contents at the phylum level. Differences were analyzed using Kruskal Wallis test; ^*^P < 0.05; *n* = 7−8 per group.

At the phylum level, the most dominant microbiota in the jejunum and ileum were Firmicutes, and those in the colon were Firmicutes and Bacteroidetes (Fig. 2A). The relative abundances of Firmicutes and Actinobacteria were lower (*P* < 0.05), whereas the relative abundances of Bacteroidetes and Spirochete were higher (*P* < 0.05) in the colon than in the jejunum or ileum (Fig. 2B−D). In the ileum, the relative abundance of Tenericutes was increased (*P* < 0.05), and Firmicutes trended to decrease (*P* = 0.06) from days 45 to 110 of gestation (Fig. 2C).

**Fig. 3:**
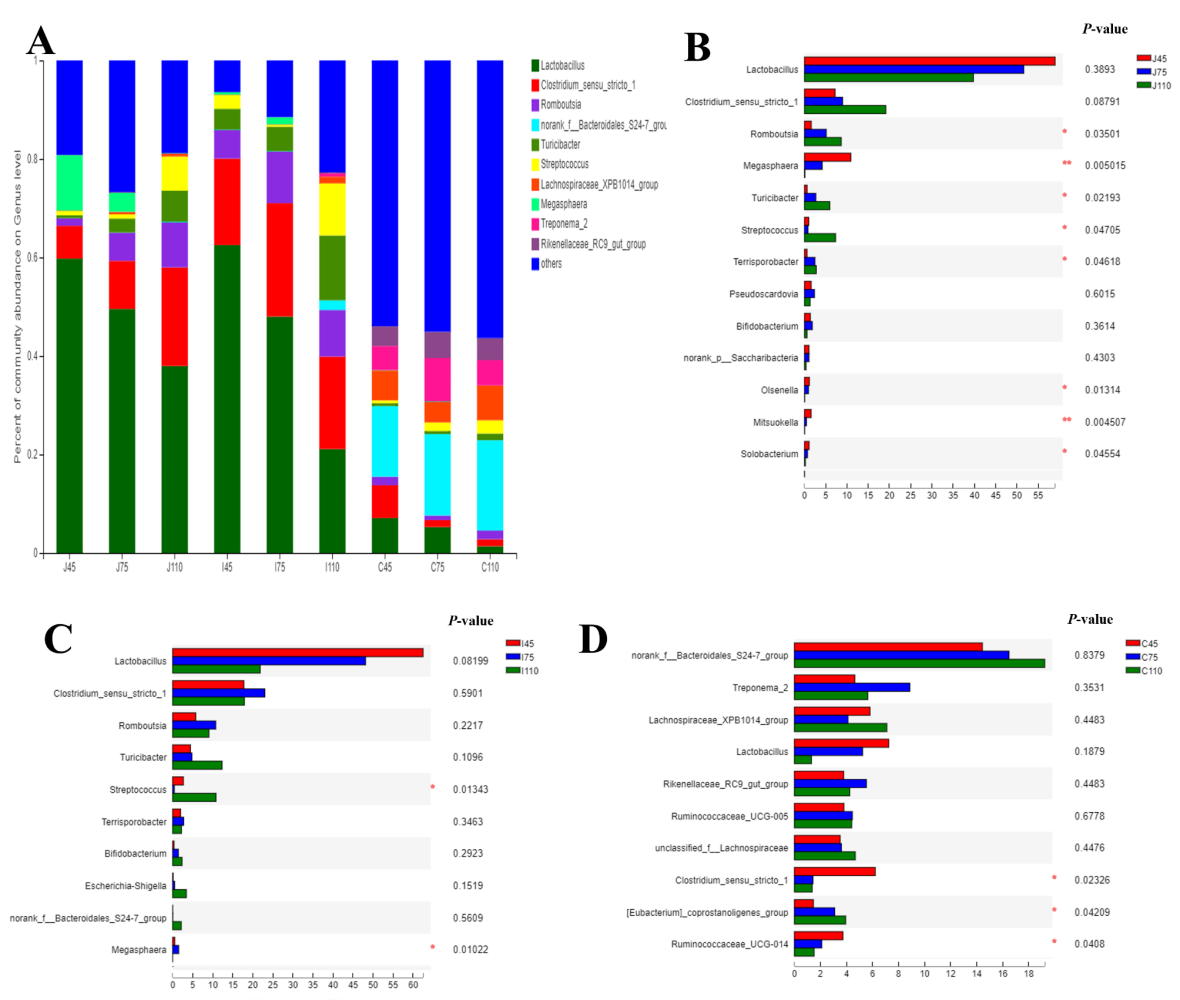
Microbiota communities at the genus level. (**A**) Relative abundance of the 10 most abundant genera in different groups. Comparison of relative abundance in jejunal (**B**), ileal (**C**), and colonic (**D**) contents at the genus level. Relative abundance of the 15 most abundant genera in different groups were listed. Differences were analyzed using Kruskal Wallis test; ^*^P < 0.05; *n* = 7−8 per group.

At the genus level, the most dominant microbiota in the jejunum and ileum were *Lactobacillus* and *Clostridium*, and those in the colon were *Bacteroidales_S24-7_group*, *Lachnospiraceae XPB1014*_*group*, and *Lactobacillus* (Fig. 3A). In the jejunum, the relative abundances of *Clostridium_sensu_stricto_1*, *Romboutsia*, *Turicibacter*, and *Streptococcus* were increased (*P* < 0.05), while the relative abundance of *Megasphaera* was decreased (*P* < 0.05) from days 45 to 110 of gestation (Fig. 3B). In the ileum, the relative abundance of *Streptococcus* was higher (*P* < 0.05) at day 110 than that at days 45 and 75 of gestation (Fig. 3C). In the colon, the relative abundances of *[Eubacterium]_coprostanoligenes_group* and *Streptococcus* were increased (*P* < 0.05), while the relative abundances of *Clostridium_sensu_stricto_1* and *Ruminococcaceae_UCG-014* were decreased (*P* < 0.05) from days 45 to 110 of gestation (Fig. 3D).

### Intestinal metabolite profiles

The results of SCFA concentrations in intestinal contents are presented in Table 1. In the jejunum, the acetate concentration was lower (*P* < 0.05) at day 75 than that at day 45 of gestation. The ileal acetate concentration increased (*P* < 0.05) linearly while the butyrate concentration decreased (*P* < 0.05) from days 45 to 110 of gestation. Moreover, the colonic acetate concentration decreased (*P* < 0.05) from days 45 to 110 of gestation.

The results of bioamine concentrations in the jejunal, ileal, and colonic contents are listed in Table 2. In the jejunum, the putrescine concentration was lower (*P* < 0.05), whereas the spermidine and spermine concentrations were higher (*P* < 0.05) at day 110 of gestation. In the ileum, the putrescine concentration was lower (*P* < 0.05) at day 110 than that at days 45 and 75 of gestation. In the colon, the cadaverine concentration was higher (*P* < 0.05) at day 45 than that at days 75 and 110 of gestation, whereas the spermine concentration was lower (*P* < 0.05) at day 110 than that at days 45 and 75 of gestation.

**Table 2:** Concentrations of bioamines in the intestinal contents in different gestation stages (µg/g, fresh contents).

Items		Day of gestation		
		45	75	110
Jejunum	Cadaverine	12.37 ± 7.76	14.91 ± 8.26	12.35 ± 7.29
	Putrescine	13.31 ± 3.46^a^	12.83 ± 5.66^a^	6.07 ± 2.56^b^
	Spermidine	4.25 ± 1.57^b^	4.15 ± 0.66^b^	6.19 ± 1.66^a^
	Spermine	4.83 ± 1.62^b^	4.36 ± 2.51^b^	8.80 ± 4.06^a^
Ileum	Cadaverine	7.16 ± 2.40	6.72 ± 1.41	5.84 ± 2.73
	Putrescine	13.38 ± 6.94^a^	14.17 ± 2.85^a^	5.70 ± 2.75^b^
	Spermidine	4.03 ± 1.76	3.00 ± 0.83	3.07 ± 1.38
	Spermine	3.73 ± 2.26	2.64 ± 1.63	2.65 ± 1.78
Colon	Cadaverine	9.09 ± 3.09^a^	5.61 ± 2.71^b^	3.55 ± 1.99^b^
	Putrescine	9.85 ± 3.78	7.88 ± 3.58	7.63 ± 3.07
	Spermidine	14.66 ± 2.65	17.10 ± 4.97	16.30 ± 3.66
	Spermine	0.94 ± 0.22^a^	1.02 ± 0.31^a^	0.46 ± 0.15^b^

Data are represented as means ± SD (*n* = 8). Different superscript lowercase letters within the same row indicate significant difference (*P* < 0.05).

### Correlation between metabolites and microbiota

**Fig. 4:**
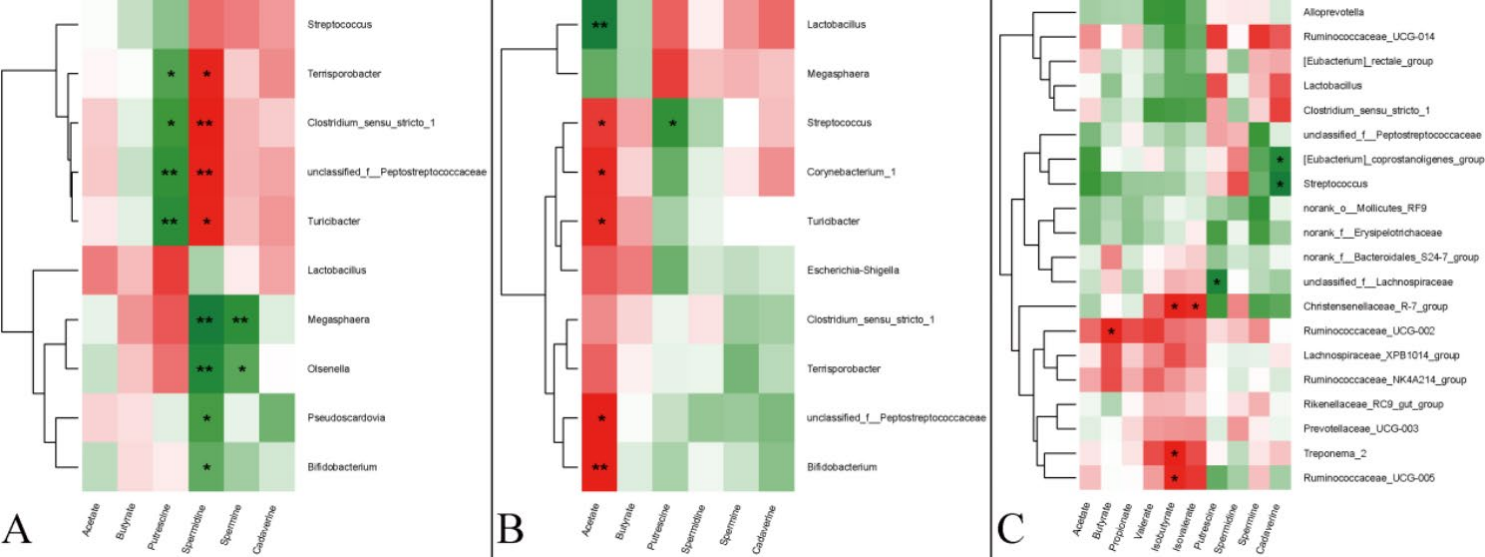
Pearson’s linear correlation heatmap of microbial metabolites and dominant genera in jejunal (**A**), ileal (**B**), and colonic (**C**) contents. ^*^ in green grid indicates a negative correlation (*P* < 0.05) between the relative abundance of the microbiota and microbial metabolites, whereas in the red grid indicates a positive correlation (*P* < 0.05).

In the jejunum (as shown in Fig. 4A), the putrescine concentration negatively correlated with the relative abundances of *Terrisporobacter, Clostridium_sensu_stricto*_*1*, and *Turicibacter*; while spermidine concentration positively correlated with the relative abundances of *Terrisporobacter*, *Clostridium sensu_stricto_1*, *Turicibacter*, and family *Peptostreptococcaceae*. The spermine concentration negatively correlated with the relative abundances of *Megasphaera* and *Olsenella*.

In the ileum (as shown in Fig. 4B), the acetate concentration positively correlated with the relative abundances of *Streptococcus*, *Corynebacterium_1*, *Turicibacter*, family *Peptostreptococcaceae*, and *Bifidobacterium*, but negatively correlated with the relative abundance of *Lactobacillus*.

In the colon (as shown in Fig. 4C), the cadaverine concentration negatively correlated with the relative abundances of *Streptococcus* and *[Eubacterium] _coprostanoligenes*_*group*. The putrescine concentration negatively correlated with the relative abundance of *unclassified*_*f_Lachnospiraceae*. The iso-butyrate concentration positively correlated with the relative abundances of *Christensenellaceae*_*R-7*_*group*, *Treponema*_*2*, and *Ruminococcaceae*_*UCG-005*. In addition, the iso-valerate concentration positively correlated with the relative abundance of *Christensenellaceae*_*R-7*_*group*.

### Function prediction of microbiota communities

**Fig. 5:**
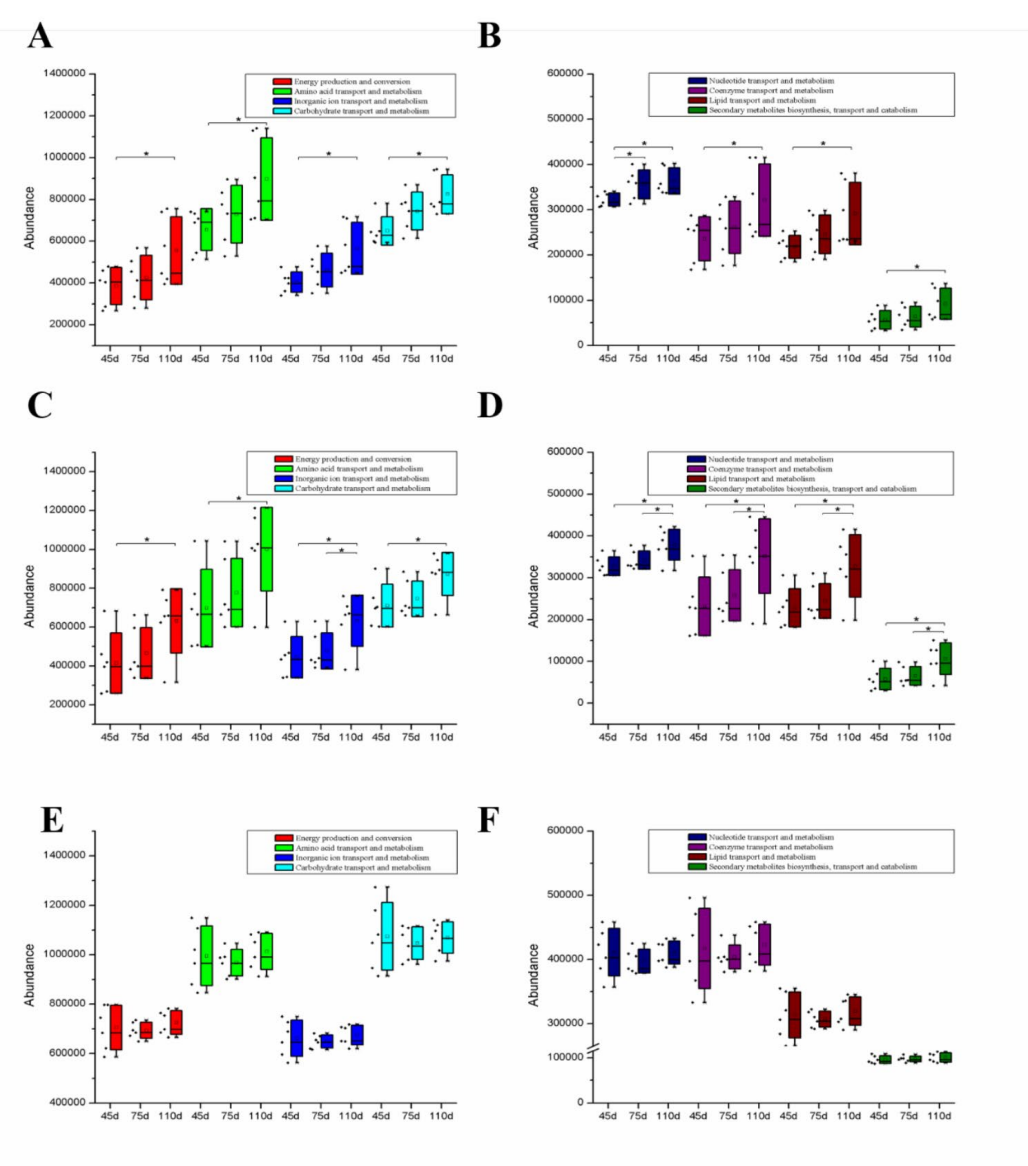
Function prediction of the microbiota communities in jejunal (**A** and **B**), ileal (**C** and **D**), and colonic (**E** and **F**) contents.

The PICRUSt1 analysis was performed to determine the metabolic function differences for jejunal and ileal microbiota. The results showed that eight metabolism functions, including energy production and conversion, amino acid transport and metabolism, inorganic ion transport and metabolism, carbohydrate transport and metabolism, nucleotide transport and metabolism, coenzyme transport and metabolism, lipid transport and metabolism, and secondary metabolites biosynthesis, transport, and catabolism were increased (*P* < 0.05) from days 45 to 110 of gestation (Fig. 5A−D). However, there was no significant change (*P* > 0.05) in metabolism functions in the colon of pigs during different gestation stages (Fig. 5E−F).

### Metabonomic analysis

**Fig. 6:**
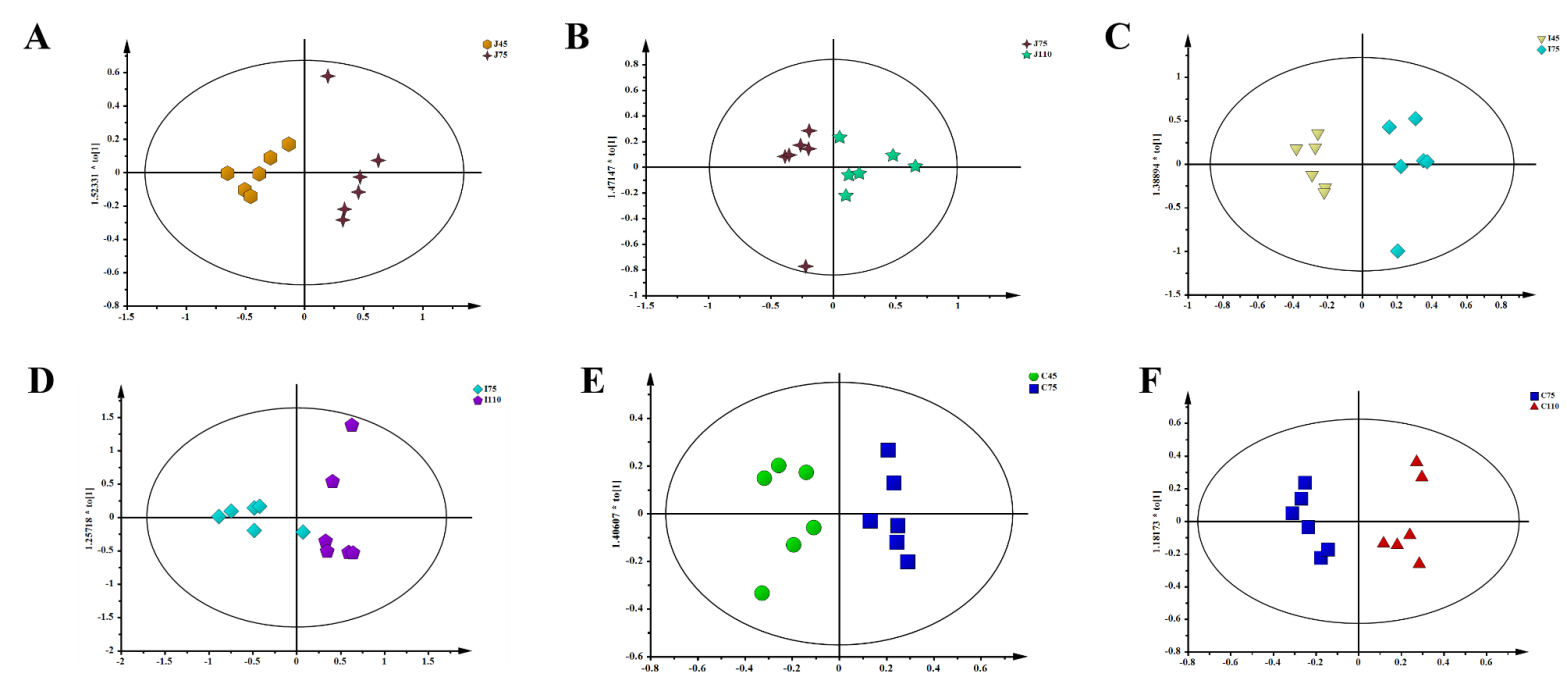
Analysis of the samples. Score scatter plots of OPLS-DA model in jejunal (**A** and **B**), ileal (**C** and **D**), and colonic (**E** and **F**) contents.

**Fig. 7:**
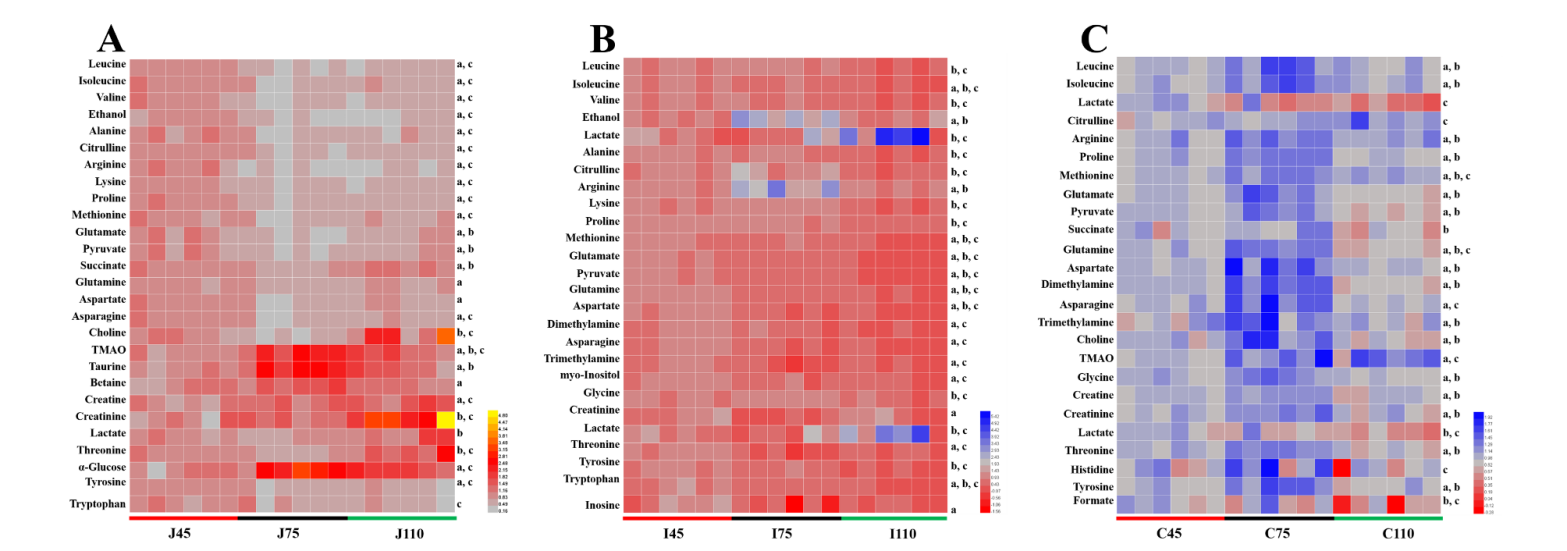
Heat-map showing the metabolites in jejunal (**A**), ileal (**B**), and colonic (**C**) contents. a−c indicated statistically significant difference (*P* < 0.05) between day 45 and day 75, day 75 and day 110, and day 45 and day 110 of gestation, respectively.

A ^1^ H NMR spectroscopy analysis was conducted to analyze the metabolite profiles in intestinal contents. The PCA score plots (Additional file 2: Fig. S3) showed distinct separation in the jejunum, ileum, and colon. The PLS-DA model showed that samples from the three gestation stages (days, 45 vs. 75 and 75 vs. 110) were well-separated (Fig. 6A−F). The concentrations of 17 metabolites, including leucine, isoleucine, valine, alanine, citrulline, arginine, lysine, proline, methionine, glutamate, pyruvate, succinate, glutamine, asparagine, choline, threonine, and tyrosine were decreased (*P* < 0.05) in the jejunum from day 45 to day 75 of gestation (Fig. 7A). In addition, the concentrations of leucine, isoleucine, valine, alanine, citrulline, arginine, lysine, proline, methionine, and tryptophan were decreased (*P* < 0.05) at day 45 of gestation compared to those at day 110 of gestation (Fig. 7A). From days 45 to 110 of gestation, the concentrations of isoleucine, methionine, glutamate, glutamine, pyruvate, and aspartate were decreased (*P* < 0.05) in the ileum (Fig. 7B). The concentrations of leucine, isoleucine, arginine, proline, methionine, glutamate, pyruvate, glutamine, aspartate, asparagine, choline, glycine, creatine, histidine, and tyrosine were increased (*P* < 0.05) in the colon from day 75 to day 110 of gestation (Fig. 7C).

### Metabolic pathway analysis

**Fig. 8:**
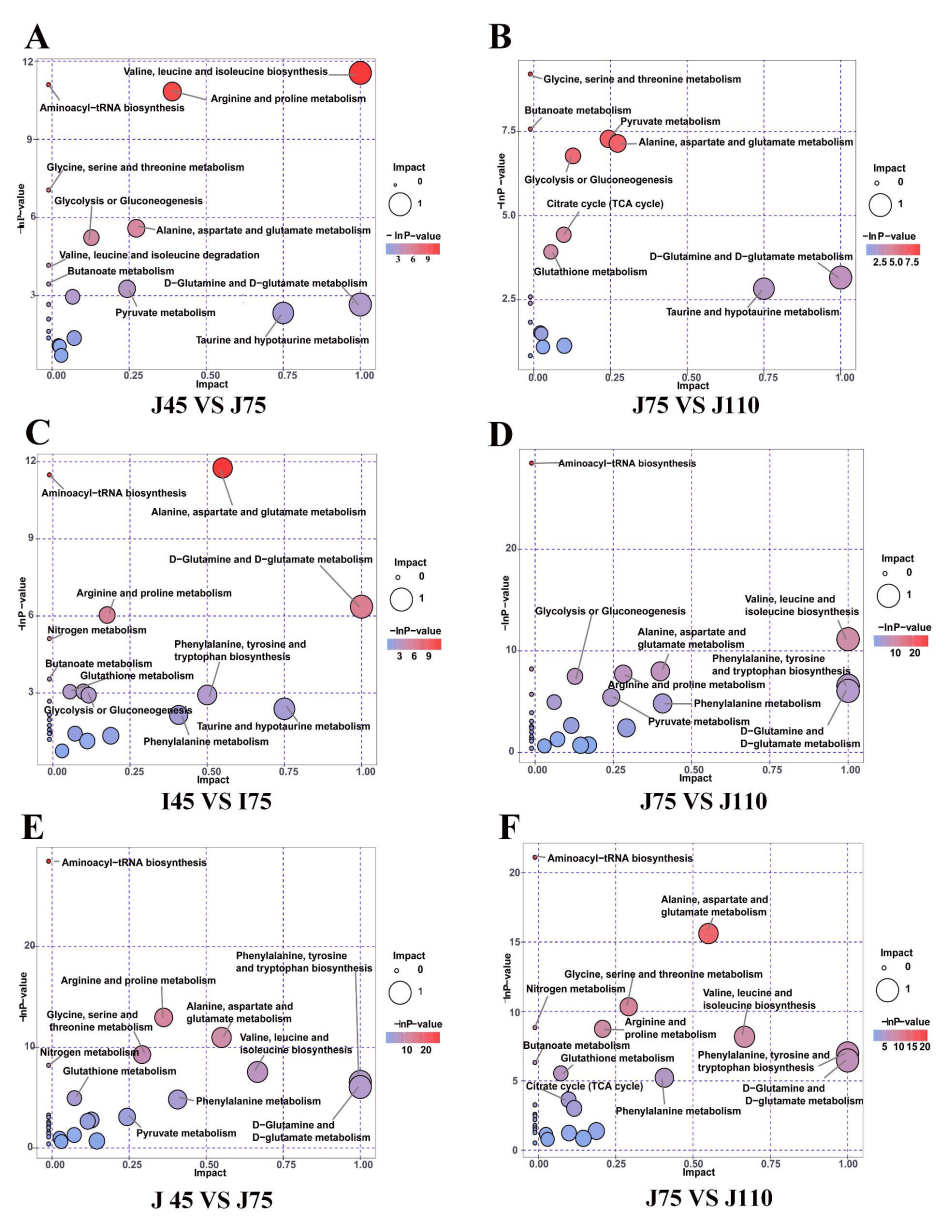
Metabolism pathway enrichment analysis among the jejunal (**A**, **B**), ileal (**C**, **D**), and colonic (**E**, **F**) contents. The X-axis shows the pathway impact, and the Y-axis provides the pathway enrichment. The size and color of the bubble indicate pathway enrichment and impact values.

MetaboAnalyst 4.0 was performed to explore the metabolic pathways in intestinal contents from early to late gestation stages. The identified metabolites with *P* < 0.05 and VIP > 1 were used to perform pathway analysis (Additional file 1: Tables S2−4). A series of metabolic pathways were affected by the gestation stage. In the jejunum, the pathways with significant interferences were D-glutamine and D-glutamate metabolism, taurine and hypotaurine metabolism, alanine/aspartate/glutamate metabolism, and pyruvate metabolism (Fig. 8A−B). The primary metabolic pathways impacted in the ileum were D-glutamine and D-glutamate metabolism, phenylalanine/tyrosine/tryptophan biosynthesis, glycolysis or gluconeogenesis, alanine/aspartate/glutamate metabolism, and arginine and proline metabolism (Fig. 8C−D), and those in the colon were D-glutamine and D-glutamate metabolism, phenylalanine/tyrosine/tryptophan biosynthesis, valine/leucine/isoleucine biosynthesis, and alanine/aspartate/glutamate metabolism (Fig. 8E−F).

## Discussion

Gut microbiota and metabolites exert important roles in maternal metabolism and physiology [17, 18]. Moreover, gut microbiota and metabolites are also associated with the changes of maternal physiology during pregnancy and play important roles in the growth and development of the fetus, and thus influence the offspring’s health later in life [19]. Therefore, the present study was conducted to investigate the changes in gut microbiota composition and microbial metabolites in sows from early (day 45) to late (day 110) gestation stages. We found that the microbiota richness and diversity in the jejunum and ileum increased from days 45 to 110 of gestation and identified several bacteria and metabolites using a microbiota-metabolome analysis. Moreover, our findings also demonstrated that acetate concentration increased in the ileum but decreased in the colon from days 45 to 110 of gestation. These findings suggested that gestation stages markedly altered the intestinal microbial community and metabolic profiles, which might be involved in maternal physiological and biological changes at late gestation and long-term health problem in the offspring.

The higher microbial richness and diversity are generally considered to be beneficial for the overall health and productivity of animals. In the present study, the richness and diversity of jejunal and ileal microbiota increased from days 45 to 110 of gestation, as evidenced by the ACE, Chao1, and Simpson indices. However, gestation stages exerted no effects on colonic microbiota richness, with no differences observed in the ACE and Chao1 indices. These findings were in line with a previous study, which reported that the gestation stage did not alter colonic microbiota richness in gestating sows [13]. By contrast, several studies showed that the diversity and richness of fecal microbiota were lower at the end than those at the beginning of pregnancy [11, 20]. This discrepancy might be explained partially by differences in the animal model; however, further studies are needed to confirm the exact reason. The animal model used in the present study was the sow, whereas that used in studies of Koren et al. [11] and Kennedy et al. [20] were human and rat, respectively. The PLS-DA showed that the microbiota composition in jejunal and ileal contents at days 45, 75, and 110 of gestation were clustered together but separated from the colonic samples, suggesting that the small intestine and colon showed different microbiota composition. In addition, the colon samples were separated in a time-dependent manner, indicating that microbiota composition in the colon presented enormous dynamic changes throughout the gestation.

At the phylum level, Firmicutes and Bacteroidetes are the most abundant bacteria in porcine intestines. In the present study, Firmicutes was the most dominant phyla in jejunal and ileal contents, and those in colonic contents were Firmicutes and Bacteroidetes, which is consistence with previous studies [12, 13]. Firmicutes and Bacteroidetes are also the dominant phyla in colonic contents in Large × Landrace pig [21]. In addition, a lower Firmicutes relative abundance and Firmicutes/Bacteroidetes ratio were observed in the ileum from days 45 to 110 of gestation, which is in agreement with the changes in body fat percentage of sows [22]. However, earlier studies showed that obesity was associated with the increased Firmicutes relative abundance and Firmicutes/Bacteroidetes ratio [23, 24]. This discrepancy might be explained by differences in the physiology of animals, diets, and tested samples.

The small intestine and colon also showed a marked differences in microbiota type at the genus level. The most dominant genera in jejunal and ileal contents were *Lactobacillus* and *Clostridium*, which in colonic contents was *norank_f_Bacteroidetes_S24-7_group*. *In vitro* and *in vivo* studies showed that *Lactobacillus* could reduce fat storage [25, 26] and protect animals against obese-insulin resistance [27]. In addition, colonization of *Lactobacillus* in the intestine was beneficial to health and prevented the colonization of opportunistic pathogens [28]. In the present study, the relative abundance of *Lactobacillus* reduced in the jejunum and ileum ongoing with pregnancy, which indicating that *Lactobacillus* was associated with changed maternal metabolism during the pregnancy. Obese women had a higher number of *Streptococcus mutants* compared to normal-weight women [29], and higher *Streptococcus* colonization was also significantly associated with obesity in children [30]. Here, the higher relative abundance of *Streptococcus* in the jejunum, ileum, and colon at day 110 of gestation suggested that *Streptococcus* might contribute to maternal fat accumulation. However, the mechanism still needs further investigation. The increase of the relative abundance of *Clostridium_sensu_stricto_1* along with the pregnancy may have connections with the inflammation in the jejunum and ileum [31]. Other studies also showed that some clusters of *Clostridium* produced butyrate and played a role in anti-inflammation [32, 33]. Based on these previous studies, we suspected that the increased relative abundance of *Clostridium_sensu_stricto_1* in the jejunum might contribute to the improvement of jejunal health as gestation extended.

Our previous study indicated that the SCFAs were associated with body fat accumulation [14]. Therefore, we investigated whether intestinal SCFAs concentration changed as gestation extended. The present study showed that the main SCFAs in jejunal and ileal contents were acetate and butyrate, while those in colonic contents were acetate, propionate, and butyrate. Acetate participates in energy generation and lipogenesis for all types of tissues [34]. Butyrate increases energy expenditure and prevents diet-induced fat accumulation and insulin resistance [35]. Consistent with the changes in maternal body fat percentage [24], ileal acetate concentration increased, and butyrate concentration decreased from days 45 to 110 of gestation, indicating that acetate and butyrate in the ileum might contribute to maternal fat accumulation in late pregnancy. Our previous study also showed that butyrate concentration was negatively associated with the body fat content in Duroc × Large White × Landrace pigs [14]. Based on these findings, we speculated that acetate and butyrate might be the main SCFAs involved in maternal fat metabolism during pregnancy.

The gestation stage also leads to different intestinal microbial functions and metabolite profiles. In the present study, microbiota in the small intestine and colon showed different functions, with eight functions increased in the jejunum and ileum, whereas these functions showed no differences in the colon. This discrepancy might be explained by differences in microbiota composition. The most dominant genera in the jejunum and ileum were *Lactobacillus* and *Clostridium*, whereas those in the colon were *norank_f_Bacteroidetes_S24-7_group*, *Treponema_2*, and *Lachnospiraceae_XPB1014*_*group*.

To investigate the functional states of gut microbiota, a metabolomics analysis was conducted in the present study. The PLS-DA analysis showed a clear cluster of metabolic profiles between different gestation stages, indicating significant differences in metabolite profiles from days 45 to 110 of gestation. At day 75 of gestation, the concentrations of amino acids were lower in the jejunum. Consistent with the changes in the jejunum, the concentrations of amino acids were decreased in the ileum as gestation extended. The main role of the jejunum and ileum is nutrient digestion and absorption. The decreased amino acid concentrations partly indicated that the digestion and absorption function of the maternal small intestine improved as gestation extended. Our results also indicate that the most influenced metabolic pathways in the intestine are D-glutamine and D-glutamate metabolism. The fetus grows quickly from day 75 of gestation to late gestation, and the mother needs to obtain adequate nutrients at this stage to support the growth and development of the fetus [7]. Therefore, we suspected that the increased metabolism functions of microbiota during late pregnancy might help the mother acquire extra nutrients. Collectively, our study showed that gut metabolites changed dramatically from early to late pregnancy, which might be associated with maternal physiology.

## Conclusion

In summary, the present study indicated that the jejunal and ileal microbiota community increased from days 45 to 110 of gestation. As gestation extended, ileal acetate concentration was increased, and butyrate concentration decreased, which were associated with the relative abundances of specific microbial genera. These findings can help us to understand the changes in gut microbiota communities and microbial metabolites from early to late pregnancy, and provide new targets in formulating the nutritional intervention to ameliorate the adverse effects of maternal fat accumulation on offspring health outcomes.

## Electronic supplementary material

Below is the link to the electronic supplementary material.

Supplementary Material 1

Supplementary Material 2

## Data Availability

The datasets supporting the conclusions of this article can be found in online repositories. The names of the repository and accession number can be found at: Science Data Bank with accession DOI: 10.57760/sciencedb.j00001.00704.
