# Supplementary material for: Dynamic alteration in the gut microbiota and metabolome of Huanjiang mini-pigs during pregnancy
Source: BMC Vet Res. 2022 Nov 3;18:385. doi: 10.1186/s12917-022-03477-0 (PMC9632071; doi:10.1186/s12917-022-03477-0)
Supplement: Supplementary file 1 — Supplementary Material 1 [file 12917_2022_3477_MOESM1_ESM.docx]

**Table S1** Ingredients and nutrient levels of the basal diet (% as-fed basis).

| Item | % |
| --- | --- |
| **Ingredients** |  |
| Corn | 54.00 |
| Soybean meal | 12.00 |
| Rice bran | 30.00 |
| Premix^1^ | 4.00 |
| Total | 100.00 |
| Nutrient levels^2^ |  |
| Digestive energy (MJ/Kg) | 13.40 |
| Crude protein | 12.04 |
| Lysine | 0.53 |
| Arginine | 0.65 |
| Proline | 0.67 |
| Ca | 0.78 |
| P | 0.62 |

^1^ Provided by per kg premix: VA 301,000 IU, VD 52,800 IU, VE 742.0 IU, VK_3_ 71.0 mg, VB_1_ 30.0 mg, VB_2_ 177.0 mg, VB_6_ 32.0 mg, VB_12_ 0.8 mg, nicotinic acid 1,073 mg, D-pantothenic acid 540.0 mg, folic acid 22.0 mg, biotin 3.0 mg, chlorine 8.0 g, Fe 2.0 g, Cu 1.0 g, Zn 3.5 g, Mn 1.3 g, I 14.0 mg, Co 35.0 mg, Se 8.3 mg, Ca 200.0 mg, and P 20.0 mg.

^2^ The values of nutrient levels were calculated value.

**Table S2** Different metabolites in jejunal contents (normalized intensity)

| ID | Compound name | J45 | J75 | J110 | VIP | | | *P* value | | |
| --- | --- | --- | --- | --- | --- | --- | --- | --- | --- | --- |
|  |  |  |  |  | J45/J75 | J45/J110 | J75/J110 | J45/J75 | J45/J110 | J75/J110 |
| 1 | Leucine | 0.056 | 0.038 | 0.039 | 1.434 | 1.464 | 0.008 | 0.001 | 0.001 | 0.808 |
| 2 | Isoleucine | 0.024 | 0.018 | 0.018 | 1.156 | 1.384 | 0.125 | 0.018 | 0.002 | 0.993 |
| 3 | Valine | 0.033 | 0.022 | 0.022 | 1.435 | 1.546 | 0.032 | <0.001 | <0.001 | 0.997 |
| 4 | Ethanol | 0.049 | 0.030 | 0.031 | 1.321 | 1.337 | 0.587 | 0.001 | 0.001 | 0.657 |
| 5 | Lactate | 0.086 | 0.085 | 0.095 | 0.099 | 0.411 | 0.642 | 0.848 | 0.577 | 0.522 |
| 6 | Alanine | 0.080 | 0.054 | 0.066 | 1.235 | 0.828 | 0.738 | 0.015 | 0.160 | 0.192 |
| 7 | Citrulline | 0.027 | 0.022 | 0.020 | 1.003 | 1.463 | 0.387 | 0.097 | <0.001 | 0.634 |
| 8 | Arginine | 0.047 | 0.026 | 0.028 | 1.386 | 1.423 | 0.489 | 0.001 | 0.001 | 0.641 |
| 9 | Lysine | 0.089 | 0.060 | 0.069 | 1.478 | 1.303 | 1.188 | <0.001 | 0.003 | 0.145 |
| 10 | Proline | 0.042 | 0.030 | 0.034 | 1.425 | 1.314 | 0.686 | 0.002 | 0.005 | 0.195 |
| 11 | Methionine | 0.018 | 0.014 | 0.015 | 0.994 | 0.701 | 0.810 | 0.031 | 0.140 | 0.315 |
| 12 | Glutamate | 0.036 | 0.022 | 0.032 | 1.280 | 0.398 | 1.710 | 0.005 | 0.425 | 0.002 |
| 13 | Pyruvate | 0.006 | 0.004 | 0.005 | 1.268 | 0.280 | 1.753 | 0.006 | 0.555 | 0.001 |
| 14 | Succinate | 0.004 | 0.004 | 0.005 | 0.438 | 1.015 | 1.219 | 0.424 | 0.043 | 0.019 |
| 15 | Glutamine | 0.021 | 0.018 | 0.021 | 0.853 | 0.053 | 0.904 | 0.121 | 0.998 | 0.194 |
| 16 | Aspartate | 0.010 | 0.008 | 0.009 | 0.721 | 0.575 | 0.583 | 0.134 | 0.239 | 0.368 |
| 17 | Dimethylamine | 0.001 | 0.001 | 0.001 | 0.373 | 0.112 | 0.003 | 0.583 | 0.825 | 0.518 |
| 18 | Asparagine | 0.006 | 0.004 | 0.005 | 0.808 | 1.106 | 0.309 | 0.129 | 0.028 | 0.566 |
| 19 | Trimethylamine | 0.001 | 0.001 | 0.001 | 0.141 | 0.081 | 0.135 | 0.843 | 0.954 | 0.893 |
| 20 | Choline | 0.011 | 0.007 | 0.022 | 1.001 | 1.095 | 1.522 | 0.035 | 0.068 | 0.032 |
| 21 | TMAO | 0.013 | 0.035 | 0.023 | 1.453 | 1.210 | 1.545 | <0.001 | 0.005 | 0.020 |
| 22 | Myo-Inositol | 0.009 | 0.008 | 0.010 | 0.311 | 0.203 | 0.582 | 0.553 | 0.549 | 0.122 |
| 23 | Taurine | 0.052 | 0.146 | 0.085 | 1.469 | 0.986 | 1.695 | <0.001 | 0.026 | 0.007 |
| 24 | Glycine | 0.041 | 0.034 | 0.033 | 0.290 | 0.535 | 0.191 | 0.372 | 0.325 | 0.895 |
| 25 | Betaine | 0.016 | 0.030 | 0.022 | 1.334 | 0.940 | 1.350 | 0.004 | 0.048 | 0.030 |
| 26 | Creatine | 0.010 | 0.017 | 0.018 | 1.388 | 1.462 | 0.103 | 0.004 | <0.001 | 0.765 |
| 27 | Creatinine | 0.004 | 0.006 | 0.013 | 1.106 | 1.447 | 1.581 | 0.041 | 0.001 | 0.016 |
| 28 | Lactate | 0.032 | 0.033 | 0.048 | 0.174 | 1.215 | 1.696 | 0.717 | 0.025 | 0.047 |
| 29 | Threonine | 0.013 | 0.011 | 0.022 | 0.609 | 1.188 | 1.709 | 0.220 | 0.042 | 0.026 |
| 30 | α-glucose | 0.036 | 0.110 | 0.074 | 1.405 | 1.278 | 1.299 | 0.001 | 0.002 | 0.052 |
| 31 | Fumarate | <0.001 | <0.001 | <0.001 | 0.547 | 0.452 | 1.049 | 0.444 | 0.525 | 0.314 |
| 32 | Histidine | 0.002 | 0.002 | 0.001 | 0.158 | 0.540 | 0.739 | 0.890 | 0.359 | 0.280 |
| 33 | Tyrosine | 0.010 | 0.008 | 0.007 | 0.727 | 1.410 | 0.804 | 0.122 | 0.002 | 0.422 |
| 34 | Phenylalanine | 0.011 | 0.009 | 0.009 | 0.825 | 0.979 | 0.314 | 0.094 | 0.070 | 0.994 |
| 35 | Tryptophan | 0.002 | 0.002 | 0.001 | 0.142 | 1.166 | 0.869 | 0.670 | 0.027 | 0.414 |
| 36 | Inosine | <0.001 | <0.001 | <0.001 | 0.384 | 0.031 | 0.764 | 0.438 | 0.960 | 0.305 |
| 37 | Formate | <0.001 | <0.001 | <0.001 | 0.232 | 0.315 | 0.342 | 0.739 | 0.564 | 0.771 |

**Table S3** Different metabolites in ileal contents (normalized intensity)

| ID | Compound name | I45 | I75 | I110 | VIP | | | *P* value | | |
| --- | --- | --- | --- | --- | --- | --- | --- | --- | --- | --- |
|  |  |  |  |  | I45/I75 | I45/I110 | I75/I110 | I45/I75 | I45/I110 | I75I110 |
| 1 | Leucine | 0.042 | 0.041 | 0.017 | 0.064 | 1.163 | 1.373 | 0.899 | 0.001 | 0.001 |
| 2 | Isoleucine | 0.022 | 0.018 | 0.008 | 0.886 | 1.267 | 1.365 | 0.118 | <0.001 | 0.001 |
| 3 | Valine | 0.030 | 0.026 | 0.011 | 0.574 | 1.232 | 1.394 | 0.330 | <0.001 | 0.001 |
| 4 | Ethanol | 0.025 | 0.055 | 0.022 | 1.529 | 0.137 | 1.299 | 0.003 | 0.648 | 0.005 |
| 5 | Lactate | 0.169 | 0.179 | 0.419 | 0.012 | 0.839 | 0.963 | 0.873 | 0.042 | 0.043 |
| 6 | Alanine | 0.068 | 0.063 | 0.027 | 0.290 | 1.235 | 1.275 | 0.573 | <0.001 | 0.006 |
| 7 | Citrulline | 0.016 | 0.022 | 0.010 | 0.939 | 0.861 | 1.178 | 0.130 | 0.033 | 0.009 |
| 8 | Arginine | 0.017 | 0.043 | 0.015 | 1.537 | 0.177 | 1.244 | 0.005 | 0.638 | 0.002 |
| 9 | Lysine | 0.065 | 0.072 | 0.030 | 0.592 | 1.140 | 1.358 | 0.367 | 0.001 | 0.001 |
| 10 | Proline | 0.032 | 0.034 | 0.016 | 0.548 | 1.186 | 1.362 | 0.445 | <0.001 | 0.001 |
| 11 | Methionine | 0.019 | 0.014 | 0.007 | 1.251 | 1.282 | 1.280 | 0.014 | <0.001 | 0.003 |
| 12 | Glutamate | 0.051 | 0.033 | 0.014 | 1.348 | 1.286 | 1.253 | 0.004 | <0.001 | 0.007 |
| 13 | Pyruvate | 0.008 | 0.005 | 0.002 | 1.383 | 1.292 | 1.211 | 0.003 | <0.001 | 0.009 |
| 14 | Succinate | 0.009 | 0.005 | 0.003 | 0.584 | 0.736 | 0.710 | 0.247 | 0.072 | 0.129 |
| 15 | Glutamine | 0.020 | 0.013 | 0.008 | 1.458 | 1.257 | 1.039 | 0.003 | <0.001 | 0.025 |
| 16 | Aspartate | 0.011 | 0.007 | 0.004 | 1.409 | 1.262 | 0.885 | 0.006 | <0.001 | 0.034 |
| 17 | Dimethylamine | 0.001 | 0.001 | <0.001 | 1.137 | 1.244 | 0.770 | 0.027 | 0.002 | 0.173 |
| 18 | Asparagine | 0.006 | 0.003 | 0.002 | 1.484 | 1.271 | 0.318 | 0.002 | <0.001 | 0.488 |
| 19 | Trimethylamine | 0.001 | <0.001 | <0.001 | 1.456 | 1.208 | 0.252 | 0.003 | 0.001 | 0.658 |
| 20 | Choline | 0.010 | 0.008 | 0.007 | 0.413 | 0.428 | 0.053 | 0.549 | 0.492 | 0.823 |
| 21 | TMAO | 0.006 | 0.009 | 0.014 | 1.047 | 0.551 | 0.430 | 0.039 | 0.325 | 0.500 |
| 22 | Myo-inositol | 0.014 | 0.008 | 0.006 | 1.191 | 1.252 | 0.678 | 0.024 | <0.001 | 0.301 |
| 23 | Taurine | 0.019 | 0.030 | 0.053 | 1.102 | 0.575 | 0.441 | 0.027 | 0.309 | 0.485 |
| 24 | Glycine | 0.043 | 0.049 | 0.026 | 1.033 | 0.833 | 1.089 | 0.117 | 0.045 | 0.008 |
| 25 | Betaine | 0.011 | 0.009 | 0.013 | 0.527 | 0.250 | 0.394 | 0.415 | 0.675 | 0.490 |
| 26 | Creatine | 0.015 | 0.011 | 0.011 | 0.644 | 0.691 | 0.224 | 0.297 | 0.179 | 0.904 |
| 27 | Creatinine | 0.009 | 0.004 | 0.006 | 1.281 | 0.744 | 0.631 | 0.031 | 0.150 | 0.172 |
| 28 | Lactate | 0.075 | 0.059 | 0.141 | 0.634 | 0.757 | 1.010 | 0.363 | 0.070 | 0.030 |
| 29 | Threonine | 0.022 | 0.007 | 0.010 | 1.413 | 1.090 | 0.555 | 0.005 | 0.006 | 0.376 |
| 30 | α-glucose | 0.011 | 0.009 | 0.040 | 0.125 | 0.577 | 0.657 | 0.869 | 0.308 | 0.289 |
| 31 | Fumarate | <0.001 | <0.001 | <0.001 | 0.107 | 0.460 | 0.620 | 0.712 | 0.445 | 0.283 |
| 32 | Histidine | 0.001 | 0.001 | 0.001 | 0.300 | 0.651 | 0.885 | 0.547 | 0.221 | 0.073 |
| 33 | Tyrosine | 0.009 | 0.007 | 0.003 | 0.731 | 1.236 | 1.300 | 0.224 | <0.001 | 0.003 |
| 34 | Phenylalanine | 0.012 | 0.008 | 0.004 | 1.211 | 1.295 | 1.249 | 0.027 | <0.001 | 0.005 |
| 35 | Tryptophan | 0.001 | 0.001 | <0.001 | 1.024 | 1.147 | 1.081 | 0.073 | 0.003 | 0.015 |
| 36 | Inosine | <0.001 | <0.001 | <0.001 | 1.170 | 1.039 | 0.573 | 0.044 | 0.037 | 0.423 |
| 37 | Formate | 0.009 | 0.014 | 0.004 | 0.491 | 0.548 | 0.894 | 0.455 | 0.262 | 0.146 |

**Table S4** Different metabolites in colonic contents (normalized intensity)

| ID | Compound name | C45 | C75 | C110 | VIP | | | *P* value | | |
| --- | --- | --- | --- | --- | --- | --- | --- | --- | --- | --- |
|  |  |  |  |  | C45/C75 | C45/C110 | C75/C110 | C45/C75 | C45/C110 | C75/C110 |
| 1 | Leucine | 0.010 | 0.014 | 0.010 | 1.220 | 0.357 | 1.110 | 0.014 | 0.591 | 0.016 |
| 2 | Isoleucine | 0.007 | 0.009 | 0.007 | 1.228 | 0.511 | 1.087 | 0.005 | 0.395 | 0.016 |
| 3 | Valine | 0.044 | 0.045 | 0.046 | 0.070 | 0.257 | 0.156 | 0.970 | 0.515 | 0.558 |
| 4 | Ethanol | 0.012 | 0.014 | 0.013 | 0.880 | 0.643 | 0.476 | 0.134 | 0.474 | 0.317 |
| 5 | Lactate | 0.048 | 0.035 | 0.026 | 0.575 | 1.775 | 0.625 | 0.131 | 0.001 | 0.262 |
| 6 | Alanine | 0.051 | 0.048 | 0.048 | 0.255 | 0.195 | 0.023 | 0.727 | 0.638 | 0.951 |
| 7 | Citrulline | 0.072 | 0.077 | 0.088 | 0.373 | 1.250 | 0.731 | 0.387 | 0.074 | 0.166 |
| 8 | Arginine | 0.011 | 0.014 | 0.012 | 1.233 | 1.197 | 0.894 | 0.006 | 0.176 | 0.024 |
| 9 | Lysine | 0.031 | 0.037 | 0.032 | 1.066 | 0.266 | 0.623 | 0.015 | 0.741 | 0.198 |
| 10 | Proline | 0.017 | 0.023 | 0.017 | 1.457 | 0.305 | 1.486 | <0.001 | 0.600 | <0.001 |
| 11 | Methionine | 0.011 | 0.013 | 0.012 | 1.352 | 1.279 | 1.111 | 0.001 | 0.042 | 0.014 |
| 12 | Glutamate | 0.020 | 0.028 | 0.018 | 1.333 | 1.475 | 1.412 | 0.002 | 0.018 | 0.001 |
| 13 | Pyruvate | 0.004 | 0.005 | 0.003 | 1.243 | 1.826 | 1.399 | 0.016 | 0.001 | 0.002 |
| 14 | Succinate | 0.005 | 0.005 | 0.004 | 0.402 | 1.176 | 1.103 | 0.278 | 0.094 | 0.012 |
| 15 | Glutamine | 0.011 | 0.015 | 0.010 | 1.307 | 1.159 | 1.448 | 0.001 | 0.053 | <0.001 |
| 16 | Aspartate | 0.008 | 0.011 | 0.007 | 1.187 | 0.398 | 1.251 | 0.013 | 0.557 | 0.010 |
| 17 | Dimethylamine | 0.001 | 0.001 | 0.001 | 1.372 | 1.669 | 1.438 | 0.001 | 0.005 | <0.001 |
| 18 | Asparagine | 0.004 | 0.006 | 0.003 | 1.246 | 0.399 | 1.289 | 0.004 | 0.483 | 0.003 |
| 19 | Trimethylamine | 0.002 | 0.002 | 0.002 | 0.957 | 0.337 | 0.979 | 0.032 | 0.694 | 0.038 |
| 20 | Choline | 0.004 | 0.006 | 0.003 | 1.255 | 0.882 | 1.319 | 0.005 | 0.187 | 0.001 |
| 21 | TMAO | 0.002 | 0.003 | 0.003 | 1.060 | 1.289 | 0.032 | 0.020 | 0.060 | 0.813 |
| 22 | Myo-inositol | 0.008 | 0.009 | 0.007 | 0.213 | 0.672 | 0.651 | 0.510 | 0.275 | 0.155 |
| 23 | Taurine | 0.010 | 0.014 | 0.013 | 0.976 | 1.219 | 0.122 | 0.034 | 0.060 | 0.591 |
| 24 | Glycine | 0.009 | 0.011 | 0.008 | 1.350 | 1.014 | 1.419 | 0.002 | 0.198 | <0.001 |
| 25 | Betaine | 0.006 | 0.007 | 0.006 | 0.748 | 0.029 | 0.605 | 0.092 | 0.958 | 0.168 |
| 26 | Creatine | 0.006 | 0.008 | 0.006 | 1.366 | 0.698 | 1.312 | 0.001 | 0.332 | 0.001 |
| 27 | Creatinine | 0.003 | 0.004 | 0.003 | 1.143 | 0.219 | 1.149 | 0.007 | 0.742 | 0.007 |
| 28 | Lactate | 0.025 | 0.022 | 0.016 | 0.273 | 1.699 | 0.949 | 0.372 | 0.002 | 0.060 |
| 29 | Threonine | 0.011 | 0.014 | 0.010 | 1.328 | 0.428 | 1.364 | <0.001 | 0.449 | <0.001 |
| 30 | α-glucose | 0.006 | 0.007 | 0.009 | 0.349 | 1.330 | 0.725 | 0.447 | 0.053 | 0.196 |
| 31 | Fumarate | <0.001 | <0.001 | <0.001 | 0.465 | 0.888 | 0.442 | 0.430 | 0.193 | 0.351 |
| 32 | Histidine | 0.001 | 0.001 | 0.001 | 0.736 | 0.760 | 0.854 | 0.133 | 0.301 | 0.052 |
| 33 | Tyrosine | 0.003 | 0.004 | 0.003 | 1.314 | 0.610 | 1.297 | 0.002 | 0.405 | 0.001 |
| 34 | Phenylalanine | 0.003 | 0.004 | 0.002 | 1.177 | 1.427 | 1.271 | 0.020 | 0.032 | 0.002 |
| 35 | Tryptophan | 0.001 | 0.001 | 0.001 | 0.312 | 0.473 | 0.151 | 0.546 | 0.429 | 0.784 |
| 36 | Inosine | 0.001 | 0.001 | 0.001 | 0.942 | 0.979 | 0.005 | 0.055 | 0.116 | 0.994 |
| 37 | Formate | <0.001 | <0.001 | <0.001 | 0.009 | 1.444 | 0.825 | 0.851 | 0.037 | 0.070 |
