# Supplementary material for: Dynamic alteration in the gut microbiota and metabolome of Huanjiang mini-pigs during pregnancy
Source: BMC Vet Res. 2022 Nov 3;18:385. doi: 10.1186/s12917-022-03477-0 (PMC9632071; doi:10.1186/s12917-022-03477-0)
Supplement: Supplementary file 2 — Supplementary Material 2 [file 12917_2022_3477_MOESM2_ESM.docx]

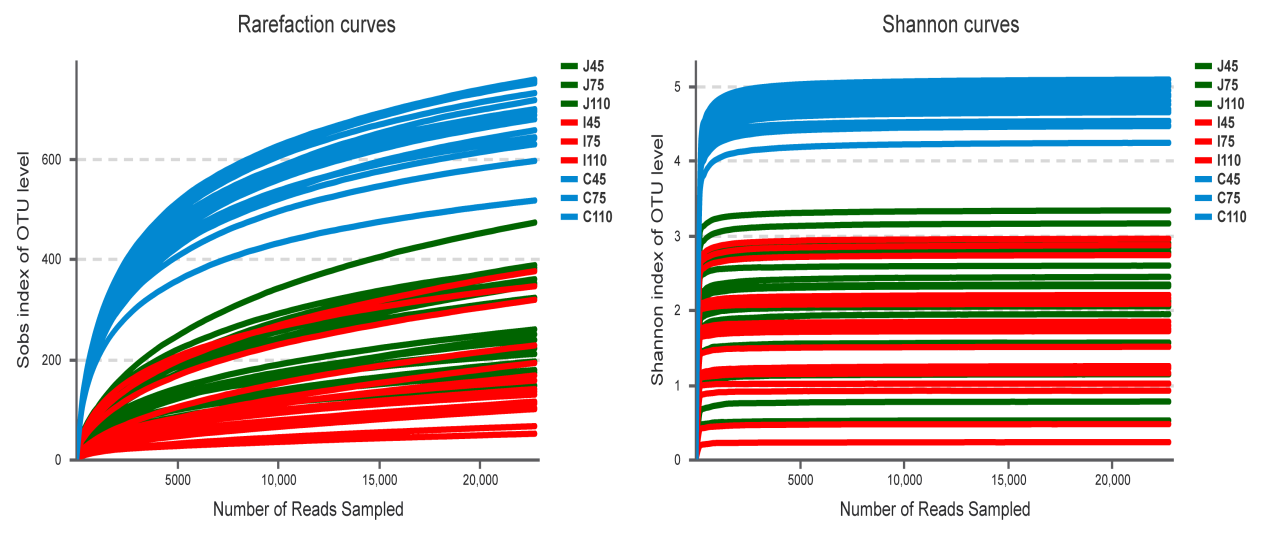


**Fig. S1** Rarefaction curves and Shannon curves at OTU level for samples. J45, J75, and J110 indicate the jejunal contents collected at days 45, 75, and 110 of gestation, respectively. I45, I75, and I110 indicate the ileal contents collected at days 45, 75, and 110 of gestation, respectively. C45, C75, and C110 indicate the colonic contents collected at days 45, 75, and 110 of gestation, respectively.



**Fig. S2** Venn diagrams of the microbiota communities of intestinal contents at OTU level. J45, J75, and J110 represent jejunal contents collected at days 45, 75, and 110 of gestation, respectively. I45, I75, and I110 represent ileal contents collected at days 45, 75, and 110 of gestation, respectively. C45, C75, and C110 represent colonic contents collected at days 45, 75, and 110 of gestation, respectively.


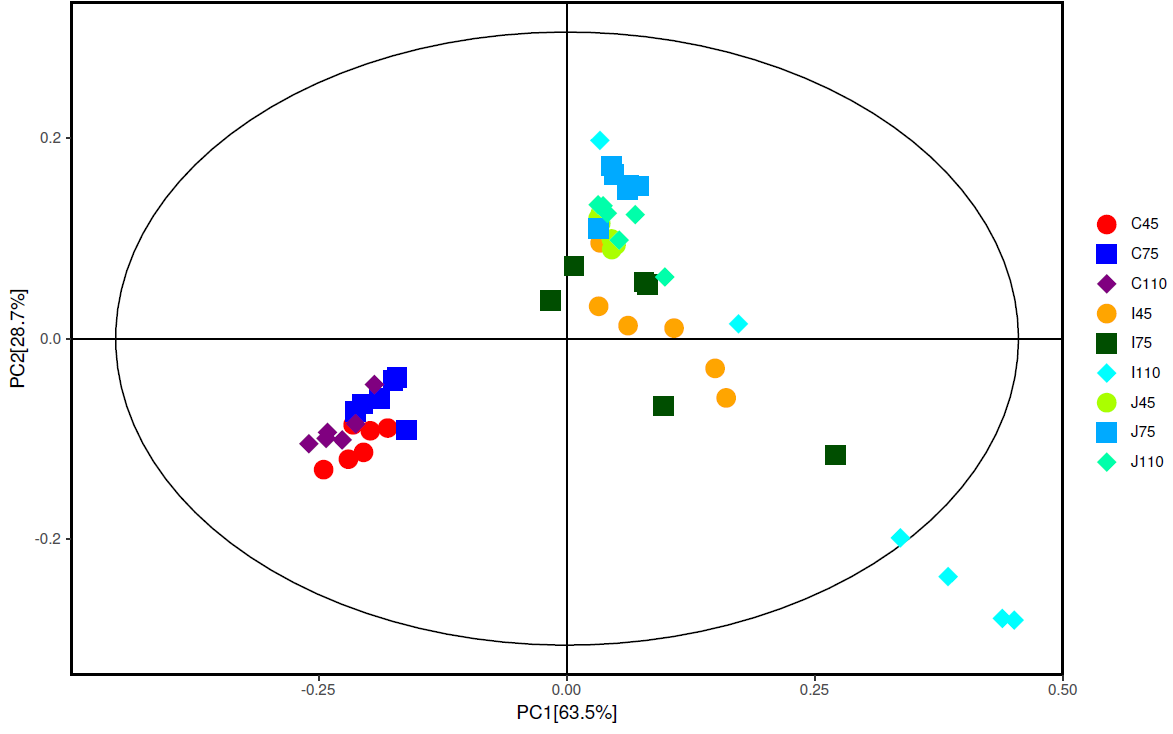


**Fig. S3** Score scatter plots of PCA model for samples. J45, J75, and J110 indicate the jejunal contents collected at days 45, 75, and 110 of gestation, respectively. I45, I75, and I110 indicate the ileal contents collected at days 45, 75, and 110 of gestation, respectively. C45, C75, and C110 indicate the colonic contents collected at days 45, 75, and 110 of gestation, respectively.
